# Association of Pre-Eclampsia with Intraoperative Hemodynamics and Postoperative Complications in Cesarean Delivery Under General Anesthesia: A Retrospective Cohort Study

**DOI:** 10.3390/jcm15020653

**Published:** 2026-01-14

**Authors:** Won Kee Min, Sejong Jin, Yongki Lee, Jeongun Cho, Sunwoo Kim, Eunsu Choi

**Affiliations:** 1 Emergency Department, Cheongsong County Health and Medical Center, Cheongsong 37433, Republic of Korea; wonkeemin@gmail.com; 2 Department of Anesthesiology and Pain Medicine, Korea University Ansan Hospital, College of Medicine, Korea University, Ansan 15588, Republic of Korea; 3 Department of Anesthesia, University of Iowa Hospitals and Clinics, Iowa City, IA 52242, USA; 4 Department of Anesthesiology and Pain Medicine, College of Medicine, Korea University, Seoul 02841, Republic of Korea

**Keywords:** pre-eclampsia, general anesthesia, cesarean section, hemodynamics, postoperative complications, respiratory complications

## Abstract

**Background:** Pre-eclampsia causes endothelial dysfunction and altered vascular reactivity, which may increase perioperative risk, particularly under the physiologic stress of general anesthesia (GA). However, the evidence regarding its independent effects under uniform GA conditions is limited. This study assessed the association between pre-eclampsia and intraoperative hemodynamic stability as well as postoperative complications in women undergoing cesarean section under GA. **Methods:** This retrospective cohort study screened 1242 women who underwent GA for cesarean delivery between January 2017 and July 2024. After applying exclusion criteria, 959 patients were included: 169 with and 790 without pre-eclampsia. The intraoperative blood-pressure and heart-rate trends, vasopressor use, operative variables, and postoperative complications were analyzed. Predictors of postoperative respiratory complications were identified using logistic regression with Firth correction. **Results:** Patients with pre-eclampsia showed consistently higher mean arterial pressures throughout induction and emergence, whereas trends in heart rate were similar. Postoperative morbidity was higher in the pre-eclampsia group (11.8% vs. 5.3%), with increased respiratory complications (3.6% vs. 1.1%) and longer hospital stays. Pre-eclampsia independently predicted postoperative respiratory complications in univariable (odds ratio [OR] 3.27, 95% confidence interval [CI] 1.13–8.90, *p* = 0.03), multivariable (OR 3.13, 95% CI 1.09–8.98, *p* = 0.03), and Firth’s analyses (OR 3.21, 95% CI 1.11–8.77, *p* = 0.03). **Conclusions:** Pre-eclampsia was associated with persistent intraoperative hypertension and higher risks of postoperative respiratory morbidity under GA. These findings support the need for individualized hemodynamic control, cautious fluid management, and increased postoperative respiratory surveillance in patients with pre-eclampsia.

## 1. Introduction

Preeclampsia, a pregnancy-specific multisystem disorder characterized by new-onset hypertension and organ dysfunction after 20 weeks of gestation [1], affects approximately 5–8% of pregnancies and remains a leading cause of maternal and perinatal morbidity and mortality worldwide [2,3]. Endothelial dysfunction, vasospasm, and increased vascular permeability result in hemodynamic instability, pulmonary edema, hepatic and renal impairments, and coagulation abnormalities [4,5,6]. These systemic derangements create distinct challenges for anesthetic management during cesarean delivery, because patients often exhibit exaggerated cardiovascular responses and impaired volume regulation [7,8].

Cesarean delivery is more common in women with pre-eclampsia because of maternal or fetal indications [9]. Neuraxial anesthesia is usually preferred owing to its favorable hemodynamic stability and avoidance of airway manipulation [10,11]. However, general anesthesia (GA) remains unavoidable in a subset of patients when regional anesthesia is contraindicated or fails or when urgent delivery is needed [12]. Under GA, laryngoscopy and intubation may provoke marked hypertensive responses, while airway edema and altered fluid balance further complicate perioperative management in patients with pre-eclampsia [13,14]. Consequently, anesthetic management in this setting is particularly demanding, and GA represents a physiologically stressful condition in which the cardiovascular and pulmonary vulnerabilities associated with pre-eclampsia may be most pronounced [15].

Most previous studies in this population have focused on comparing maternal and neonatal outcomes between neuraxial anesthesia and GA, consistently emphasizing the benefits of neuraxial techniques [16,17,18]. However, relatively few investigations have examined the independent perioperative impact of pre-eclampsia itself under uniform anesthetic conditions, particularly in patients managed with GA. Many prior reports were limited by small samples, heterogeneous settings, and confounding factors between disease severity and anesthetic choice, making it difficult to isolate the specific contribution of preeclampsia [16,18]. Accordingly, detailed evaluations of intraoperative hemodynamic behavior and postoperative morbidity under GA remain limited [19,20]. Among these outcomes, postoperative respiratory complications merit particular attention, as they reflect the interaction between GA-related ventilatory factors [21,22] and the endothelial and fluid abnormalities characteristic of pre-eclampsia [23].

To address these gaps, this retrospective cohort study evaluated the association between pre-eclampsia and perioperative outcomes in women undergoing cesarean section under GA at a tertiary care hospital, with a focus on intraoperative hemodynamic stability and postoperative respiratory complications. Intraoperative hemodynamic variables, including blood-pressure variability and vasopressor use, as well as a range of postoperative complications—respiratory, gastrointestinal, hemorrhagic, infectious, and cardiovascular—were systematically assessed. We hypothesized that pre-eclampsia would be associated with greater intraoperative hemodynamic variability and an increased overall risk of postoperative complications.

These findings have potential implications for perioperative management in high-risk obstetric populations. Recognition of the heightened hemodynamic and respiratory vulnerability of patients with pre-eclampsia under GA may inform closer intraoperative monitoring, individualized fluid management strategies, and consideration of postoperative intensive care unit observation in selected patients.

## 2. Materials and Methods

### 2.1. Study Design and Setting

This retrospective cohort study was conducted at Korea University Ansan Hospital, a tertiary referral center in Gyeonggi-do, Republic of Korea, between 1 January 2017, and 31 July 2024. We aimed to evaluate whether pre-eclampsia is associated with perioperative outcomes, specifically intraoperative hemodynamic stability and postoperative complications, in women who underwent cesarean delivery under GA. All data were retrieved from the institutional electronic medical records system. The study protocol was reviewed and approved by the Institutional Review Board of Korea University Ansan Hospital (IRB No. 2024AS0208). The requirement for informed consent was waived because of the retrospective nature of the study. This study adhered to the principles of the Declaration of Helsinki and was conducted and reported in accordance with the Strengthening the Reporting of Observational Studies in Epidemiology (STROBE) guidelines [24].

Although retrospective in nature, the study was designed as an exposure-based cohort analysis. The source population consisted of all women who underwent cesarean delivery under GA during the study period. Exposure status, defined as the presence or absence of pre-eclampsia, was determined at the time of surgery, prior to the occurrence of postoperative outcomes. All patients entered the cohort at a uniform time point—the time of cesarean delivery—and were followed through their postoperative hospital course until discharge, during which postoperative complications were systematically ascertained.

For the purpose of this study, we intentionally restricted the analysis to patients who underwent cesarean delivery under GA. GA is associated with physiologic stress related to induction, airway manipulation, and emergence, which are recognized periods of cardiovascular and pulmonary instability during anesthesia, particularly in patients with underlying maternal comorbidities. Accordingly, the study was designed to focus on a clinically relevant anesthetic context in which GA was required, allowing assessment of the perioperative effects of pre-eclampsia under uniform anesthetic conditions. In addition, this design choice minimized confounding related to anesthetic technique, which is a major determinant of intraoperative hemodynamics and postoperative outcomes in obstetric anesthesia.

### 2.2. Study Population

During the study period, a total of 1242 women underwent cesarean delivery under GA at the study institution and constituted the source population. Among them, 221 women had a diagnosis of pre-eclampsia. Women who underwent cesarean delivery under GA and had complete perioperative data, including intraoperative hemodynamic records and postoperative outcome information, were eligible for inclusion.

Exclusion criteria were predefined and included incomplete medical records, American Society of Anesthesiologists (ASA) physical status IV or higher, sepsis or hemodynamic instability at the time of surgery, and pre-existing severe systemic diseases known to independently cause hemodynamic compromise. Women with multiple gestations or those who received combined regional anesthesia and GA were also excluded to maintain a clinically homogeneous cohort.

After applying these criteria, 959 patients were included in the final cohort: 169 women with pre-eclampsia and 790 women without pre-eclampsia.

### 2.3. Data Collection and Variables

Demographic, obstetric, and clinical data were collected from medical records, including age, height, weight, body mass index, gestational age, comorbidities, and preoperative laboratory results (e.g., hemoglobin, platelet count, electrolytes, liver and renal function tests, coagulation profiles, and albumin levels). Intraoperative and peripartum variables included the type of surgery (elective or emergency), anesthetic time, operative time, estimated blood loss, total crystalloid volume administered, urine output, and neonatal Apgar scores at 1 and 5 min. Postoperative variables included total hospital stay, intensive care unit (ICU) admission, and postoperative complications, such as respiratory events, hypertension requiring additional treatment, fever, ileus, or bleeding. All postoperative complications were identified by reviewing electronic medical records, and their presence was independently verified by two investigators.

### 2.4. Definitions and Outcome Measures

Pre-eclampsia was defined according to the American College of Obstetricians and Gynecologists (ACOG) criteria as new-onset hypertension (systolic ≥ 140 mmHg or diastolic ≥ 90 mmHg) after 20 weeks of gestation accompanied by proteinuria (≥300 mg/24 h) or signs of end-organ dysfunction [1].

The study had the following two primary outcomes: intraoperative hemodynamic changes and postoperative complications. Hemodynamic variables, including the mean arterial pressure and heart rate (HR), were recorded at six standardized time points as follows: before induction, after induction, after intubation, at skin incision, during delivery, and during emergence. These data were used to generate a hemodynamic trend graph. Postoperative complications were defined as any adverse events that occurred during hospitalization after cesarean delivery. Respiratory complications included oxygen desaturation (SpO_2_ < 90%), pulmonary edema confirmed by clinical findings or chest imaging, and respiratory failure requiring supplemental oxygen or mechanical ventilation. Hypertensive events were defined as sustained blood pressure of ≥160/110 mmHg requiring pharmacological intervention.

### 2.5. Anesthetic Management

In our institution, neuraxial anesthesia is generally preferred and recommended as the first-line anesthetic technique for cesarean delivery, including in patients with pre-eclampsia, in accordance with established international guidelines [25]. GA was not used routinely, but was selected only when clinically indicated, such as in cases of urgent or emergent delivery, contraindications to neuraxial anesthesia (e.g., coagulopathy or thrombocytopenia), failed neuraxial block, or when rapid delivery was required [1,26,27].

All general anesthetic procedures were performed by board-certified anesthesiologists or supervised anesthesia residents according to a standardized institutional protocol. Anesthesia was induced with intravenous agents (propofol or thiopental) and maintained with volatile anesthetics (sevoflurane or desflurane) in an oxygen–nitrous oxide mixture. Rocuronium was administered for neuromuscular blockade and opioids such as fentanyl or remifentanil were titrated as needed. Noninvasive blood pressure was measured at 1 min intervals, and vasoactive drugs (ephedrine, phenylephrine, nicardipine, or labetalol) were used as clinically indicated. After delivery, oxytocin was administered, and the anesthetic agents were gradually reduced before emergence. Postoperative care was provided in the recovery room, followed by the patient being transferred to the obstetric ward. No predefined group-specific dosing adjustments were applied based on pre-eclampsia status; induction agents, opioid administration, and anesthetic management after umbilical cord clamping were guided by institutional practice and intraoperative clinical judgment rather than by disease group.

### 2.6. Statistical Analysis

Continuous variables were expressed as mean ± standard deviation (SD) or median (interquartile range [IQR]). The normality of continuous variables was assessed using the Shapiro–Wilk test to determine the appropriate use of parametric or nonparametric tests. Group comparisons were performed using Student’s t-test or Mann–Whitney U-test, as appropriate. Categorical variables are expressed as numbers and percentages and were compared using the chi-square or Fisher’s exact test. Univariable and multivariable logistic regression analyses were performed to identify factors associated with postoperative respiratory complications. Maternal age and diabetes mellitus were included as covariates in the regression model because both have been reported to be established risk factors for postoperative pulmonary complications in surgical populations, reflecting an age-related decline in pulmonary reserve as well as diabetes-associated endothelial dysfunction and fluid imbalance, respectively [21,28,29]. Other perioperative variables closely related to disease manifestation or clinical decision-making were not included in the multivariable model to avoid overadjustment and to preserve model stability given the limited number of outcome events. To account for potential bias owing to the small number of events, Firth’s penalized likelihood logistic regression was conducted [30,31,32]. Results are reported as odds ratios (ORs) with 95% confidence intervals (CIs), and statistical significance was set at *p* < 0.05. All analyses were performed using IBM SPSS Statistics (version 26.0; IBM Corp., Armonk, NY, USA).

## 3. Results

### 3.1. Baseline Characteristics

**Figure 1:**
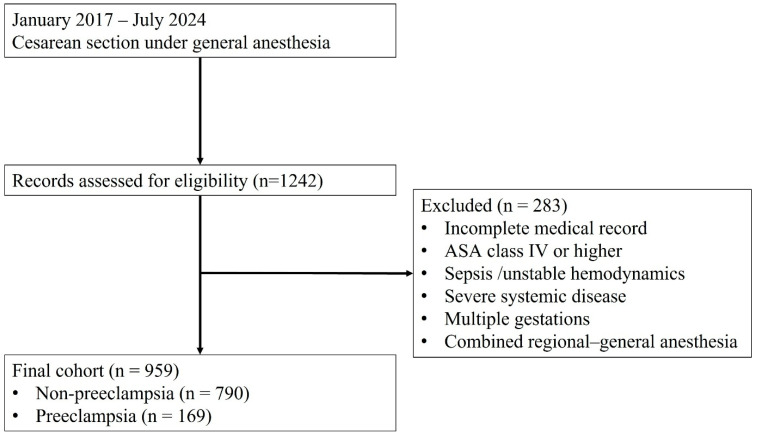
Flow diagram of patient selection for the study cohort.

During the study period (2017–2024), 1242 women underwent cesarean section under GA at our institution. Of these, 283 were excluded based on predefined criteria, including incomplete medical records, high ASA class, hemodynamic instability or sepsis, severe systemic disease, multiple gestations, and combined regional anesthesia and GA. The final cohort included 959 patients: 169 with and 790 without pre-eclampsia. Figure 1 illustrates the patient selection process.

Patient baseline characteristics are summarized in Table 1. The two groups were similar in age and height, whereas women with pre-eclampsia had higher bodyweight (*p* = 0.011). As expected, hypertension was observed in all patients with pre-eclampsia. Laboratory findings differed significantly between the groups; women with pre-eclampsia showed higher hemoglobin, AST, BUN, creatinine, and potassium levels but lower albumin levels and prolonged PT (INR) than women without pre-eclampsia (all *p* < 0.05). Abnormal chest radiographic findings were also more frequent in the pre-eclampsia group than in the non-pre-eclampsia group (*p* = 0.015). These findings suggest that women with pre-eclampsia underwent surgery with greater metabolic and vascular stress than women without pre-eclampsia.

### 3.2. Intraoperative Variables and Hemodynamic Changes

Operative details are shown in Table 2. Emergency cesarean delivery was more common and gestational age was shorter in the pre-eclampsia group than in the non-pre-eclampsia group; the operative and anesthetic durations were comparable between groups. The estimated blood loss and infused crystalloid volumes were slightly lower in patients with pre-eclampsia than in those without pre-eclampsia.

**Figure 2:**
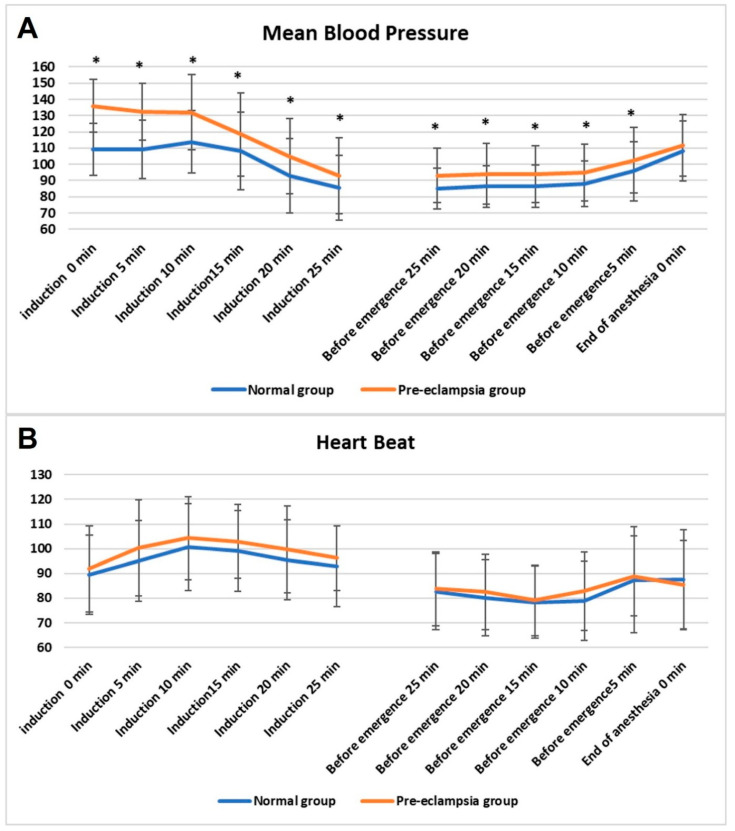
Intraoperative hemodynamic changes during cesarean delivery under general anesthesia in women with and without pre-eclampsia. (**A**) Mean arterial pressure (MAP) and (**B**) heart rate (HR) trends at predefined time points during general anesthesia in women undergoing cesarean delivery. The pre-eclampsia (orange line) and the control (blue line) groups are shown for comparison. Error bars represent standard deviations. * indicates *p* < 0.05 for between-group comparisons at the corresponding time points.

Intraoperative hemodynamic trends are shown in Figure 2A,B. The mean blood pressure was persistently higher in the pre-eclampsia group than in the non-pre-eclampsia group throughout both induction and emergence, reaching statistical significance at several time points (*p* < 0.05). The HR patterns were comparable between groups. These findings suggest that patients with pre-eclampsia maintain elevated systemic vascular resistance under GA without compensatory tachycardia, reflecting persistent vascular hyperreactivity.

### 3.3. Postoperative Outcomes

Postoperative findings are summarized in Table 3. Overall, complications occurred more frequently (11.8% vs. 5.3%) and both total and postoperative hospital stays were significantly longer in the pre-eclampsia group than in the non-pre-eclampsia group. Respiratory complications and the need for postoperative antihypertensive medication were also more frequent among women with pre-eclampsia than among those without pre-eclampsia, whereas other events such as ileus, bleeding, or fever did not differ significantly. These results indicate that the systemic vascular and fluid imbalance characteristics of pre-eclampsia may persist beyond the intraoperative period and contribute to delayed recovery.

### 3.4. Respiratory Complications and Predictive Factors

Respiratory complications demonstrated the largest between-group differences; therefore, further analysis was conducted. As shown in Table 4, patients who developed postoperative respiratory complications were more likely to have pre-eclampsia and to require ICU admission, whereas other perioperative characteristics did not differ substantially between the groups.

Logistic regression analyses identified pre-eclampsia as the only significant predictor of postoperative respiratory complications. Pre-eclampsia was associated with an approximately threefold increase in risk (univariable OR 3.27, 95% CI 1.13–8.90, *p* = 0.03). This association remained significant after adjusting for maternal age and diabetes mellitus (adjusted OR 3.13, 95% CI 1.09–8.98, *p* = 0.03). Firth’s penalized regression yielded a similar effect estimate (OR 3.21, 95% CI 1.11–8.77, *p* = 0.03), supporting the robustness of this finding despite the small number of events.

## 4. Discussion

This retrospective cohort study investigated the association between pre-eclampsia and perioperative outcomes in women undergoing cesarean delivery under GA. Pre-eclampsia was associated with persistent intraoperative hypertension and a higher incidence of postoperative morbidities, particularly respiratory complications. These findings indicate that, even under standardized anesthetic conditions, systemic pathophysiological alterations due to pre-eclampsia continue to influence perioperative outcomes.

Neuraxial anesthesia remains the recommended first-line anesthetic technique for cesarean delivery, including in patients with pre-eclampsia [25]. Accordingly, the present study does not advocate the routine use of GA, but focuses on a clinically relevant subset of patients in whom GA was required because of specific clinical indications. The findings should therefore be interpreted within the context of cesarean deliveries performed under GA.

The hemodynamic patterns observed in patients with pre-eclampsia reflect sustained vascular hyperreactivity rather than transient anesthetic responses. Endothelial dysfunction, decreased nitric oxide bioavailability, and exaggerated sympathetic activity are well-documented features of pre-eclampsia that limit vasodilatory reserves and enhance responsiveness to catecholamines [33,34,35,36,37]. Such mechanisms likely explain why patients with pre-eclampsia exhibit persistently elevated mean blood pressure during both induction and emergence despite similar HR trends, suggesting that their hypertensive response is driven primarily by vascular tone rather than cardiac output. These results are consistent with those of previous reports describing heightened pressor responses and labile hemodynamics parturients with pre-eclampsia, emphasizing that anesthetic depth alone cannot fully attenuate these vascular abnormalities [15,38,39,40].

In addition to intraoperative responses, women with pre-eclampsia demonstrated greater postoperative morbidity and prolonged hospitalization [19,41,42]. Although the absolute number of respiratory events was small, the higher incidence observed in the preeclampsia group may be of clinical relevance. These pathophysiological features of pre-eclampsia, including increased capillary permeability [43], impaired cardiac relaxation [44,45], and reduced oncotic pressure owing to hypoalbuminemia [46,47] may predispose patients to pulmonary congestion or edema in the postoperative period. Under GA, where positive pressure ventilation and fluid redistribution occur, these patients are particularly vulnerable [15]. In our analyses, the association between pre-eclampsia and postoperative respiratory complications showed a consistent direction across univariable, multivariable, and penalized regression models, suggesting that the observed relationship is unlikely to be solely attributable to model instability. This finding is consistent with population-based studies linking pre-eclampsia to pulmonary edema, cardiac dysfunction, and prolonged recovery after cesarean delivery [42,43,48]. Nonetheless, causal inference is limited by the retrospective nature of this cohort study.

However, the existing literature on perioperative and postoperative respiratory outcomes in women with pre-eclampsia has yielded heterogeneous results. While several studies have reported an increased risk of pulmonary edema or respiratory compromise associated with pre-eclampsia, particularly in the setting of cesarean delivery, other investigations have demonstrated weaker or non-significant associations [49,50,51,52].

Such inconsistencies may be attributable to substantial heterogeneity in study design, including variability in anesthetic management, the absence of stratification according to pre-eclampsia severity, and differences in the definition of respiratory outcomes. In many prior studies, general and neuraxial anesthesia were analyzed together, limiting the ability to isolate the physiological effects of pre-eclampsia under the specific stresses imposed by GA. Moreover, studies focusing primarily on overt pulmonary edema or severe maternal morbidity may have underrecognized milder but clinically relevant respiratory events, such as transient hypoxemia or postoperative oxygen requirement.

In light of these considerations, the present findings should be interpreted as complementary to, rather than contradictory with, existing evidence. By restricting the analysis to women undergoing cesarean delivery under GA and systematically assessing postoperative respiratory events during hospitalization, this study addresses a specific perioperative context in which the vascular and fluid-related abnormalities of pre-eclampsia may become more apparent. Importantly, these results do not suggest that pre-eclampsia uniformly confers an increased respiratory risk across all anesthetic settings but rather that such risk may be context-dependent and potentially amplified under GA.

Most earlier studies compared neuraxial anesthesia and GA in women with pre-eclampsia, consistently reporting superior hemodynamic stability and neonatal outcomes with regional techniques, including higher Apgar scores and reduced need for neonatal resuscitation [10,16,17]. In contrast, relatively few investigations have examined the independent perioperative impact of pre-eclampsia itself under uniform general anesthetic conditions. By minimizing confounding related to anesthetic technique, the present study contributes to this limited body of evidence and supports the concept that the vascular pathology of pre-eclampsia may persist beyond delivery and influence postoperative recovery.

Clinically, the observed increase in postoperative respiratory complications among women with pre-eclampsia undergoing GA underscores the importance of anticipatory perioperative management in this population. In the present cohort, pre-eclampsia was associated with a higher incidence of postoperative respiratory morbidity, indicating a clinically relevant vulnerability during the postoperative period. Although specific anesthetic or perioperative management strategies were not directly evaluated in this study, these findings may plausibly suggest that careful titration of induction agents, avoidance of excessive sympathetic stimulation, and preparedness for vasoactive interventions may be particularly relevant when GA is required. Similarly, fluid administration may warrant individualization to balance the competing risks of hypoperfusion and pulmonary congestion, a consideration informed by the observed postoperative respiratory vulnerability rather than by tested interventional approaches. Given the empirically observed increase in postoperative respiratory complications, enhanced postoperative surveillance in a monitored setting may reasonably be considered in selected patients with pre-eclampsia undergoing GA. Overall, these observations support a multidisciplinary perioperative approach integrating obstetric, anesthetic, and critical care perspectives, while highlighting the need for future studies to evaluate whether targeted perioperative strategies can mitigate postoperative respiratory risk in this high-risk population.

This study has several limitations that merit consideration. First, the retrospective study design inherently limits causal inference and may have introduced residual confounding from unmeasured variables, such as the severity or chronicity of pre-eclampsia, detailed intraoperative fluid composition, or anesthetic drug dosing. In particular, the lack of stratification according to pre-eclampsia severity may have resulted in an underestimation of the true association, as patients with milder disease were analyzed together with those with more severe manifestations. Second, although all patients received GA, subtle variations in anesthetic management, including the choice of volatile agents or the timing and intensity of vasoactive drug administration, were not fully standardized; such variability may have attenuated differences between groups, potentially biasing the observed associations toward the null. Third, postoperative complications were identified through review of electronic medical records, and mild or transient respiratory events may therefore have been underreported, leading to conservative estimates of postoperative respiratory morbidity. Fourth, the study was conducted at a single tertiary referral center, which may limit external validity and reflect a population with relatively severe disease or specialized perioperative care. In addition, because GA is not routinely used as a first-line anesthetic technique for cesarean delivery, particularly in patients with pre-eclampsia, the generalizability of our findings may be further constrained. Finally, the total number of postoperative respiratory events was small relative to the overall cohort size; although Firth’s penalized logistic regression was applied to mitigate small-event bias, the limited number of outcome events may still restrict the precision of the estimated effect size, as reflected by wider confidence intervals.

Despite these limitations, this study analyzed a relatively large and homogeneous cohort managed using consistent institutional protocols. The uniform use of GA minimized confounding by anesthetic technique and provided real-world evidence of how pre-eclampsia influences perioperative outcomes. These strengths enhance the reliability and clinical applicability of our findings and offer valuable insights into the anesthetic implications of pre-eclampsia.

## 5. Conclusions

Pre-eclampsia was significantly associated with sustained intraoperative hypertension and increased postoperative morbidity under GA. Meticulous intraoperative control, cautious fluid management, and vigilant postoperative monitoring may be important for improving safety in this high-risk population. Future prospective studies incorporating continuous hemodynamic monitoring, detailed fluid balance assessment, and standardized anesthetic protocols are warranted to clarify causality and further refine perioperative care strategies for women with pre-eclampsia. Such efforts may ultimately contribute to safer anesthetic practices and improved maternal outcomes in this high-risk population.

## Figures and Tables

**Table 1:** Preoperative baseline characteristics of women undergoing cesarean delivery under general anesthesia.

	Non-Pre-Eclampsia (n = 790)	Pre-Eclampsia (n = 169)	*p*-Value
**Demographics**			
Age (years)	34.2 ± 4.9	34.4 ± 5.3	0.641
Height (cm)	160.0 ± 13.0	160.3 ± 6.3	0.698
Weight (kg)	74.0 ± 34.2	80.8 ±16.3	0.011 *
**Comorbidities**			
Hypertension	29 (3.7)	169 (100.0)	<0.001 ***
Diabetes	105 (13.3)	35 (20.7)	0.016 *
Respiratory disease	18 (2.3)	5 (3.0)	0.581
Endocrine disease	49 (6.2)	12 (7.1)	0.605
Neurologic disease	9 (1.1)	3 (1.8)	0.454
**Laboratory findings**			
Hemoglobin (g/dL)	11.5 ± 1.6	12.0 ± 1.8	0.004 **
White blood cell (10^3^/μL)	15.7 ± 66.5	12.0 ± 13.9	0.506
Platelet (10^3^/μL)	225.8 ± 67.3	214.4 ± 74.1	0.078
PT (INR)	1.0 ± 0.1	0.9 ± 0.1	<0.001 ***
aPTT (sec)	30.2 ± 10.9	31.4 ± 20.3	0.389
Albumin (g/dL)	3.7 ± 1.5	3.2 ± 0.9	<0.001 ***
AST (U/L)	17.0 ± 19.8	26.0 ± 43.4	0.019 *
ALT (U/L)	16.2 ± 34.5	24.4 ± 45.3	0.053
Total bilirubin (mg/dL)	0.3 ± 0.2	0.3 ± 0.2	0.247
Glucose (mg/dL)	93.7 ± 25.2	92.2 ± 34.8	0.610
BUN (mg/dL)	8.9 ± 8.2	12.4 ± 6.2	<0.001 ***
Creatinine (mg/dL)	0.6 ± 0.7	0.7 ± 0.4	0.042 *
Sodium (mmol/L)	137.5 ± 4.8	136.3 ± 11.1	0.233
Potassium (mmol/L)	4.1 ± 0.4	4.3 ± 0.4	0.001 **
Chloride (mmol/L)	103.9 ± 2.1	104.3 ± 2.9	0.119
**Others**			
Abnormal chest X-ray finding, n (%)	7 (0.9)	6 (3.6)	0.015 *
Abnormal EKG finding, n (%)	49 (6.2)	14 (8.3)	0.298

Values are presented as number (percentage) or mean ± standard deviation. * *p* < 0.05, ** *p* < 0.01, *** *p* < 0.001.

**Table 2:** Operative and intraoperative characteristics of women undergoing cesarean delivery under general anesthesia.

	Non-Pre-Eclampsia (n = 790)	Pre-Eclampsia (n = 169)	*p*-Value
Emergency operation	464 (58.7)	128 (75.7)	<0.001 ***
Gestational days	255.9 ± 22.8	241.0 ± 27.1	<0.001 ***
Apgar score at 1 min	6.8 ± 2.2	7.0 ± 2.1	0.270
Apgar score at 5 min	8.6 ± 1.8	8.8 ± 1.4	0.942
Operation time (min)	60.4 ± 19.0	60.2 ± 13.7	0.853
Anesthetic time (min)	78.3 ± 21.2	78.0 ± 14.2	0.857
Estimated blood loss (mL)	455.6 ± 274.3	406.0 ± 226.5	0.046 *
Infused crystalloid (mL)	766.3 ± 496.5	686.2 ± 392.3	0.037 *

Values are presented as mean ± standard deviation (SD) or number (percentage). * *p* < 0.05, *** *p* < 0.001.

**Table 3:** Postoperative complications in women with and without pre-eclampsia.

	Non-Pre-Eclampsia (n = 790)	Pre-Eclampsia (n = 169)	*p*-Value
**Length of hospital stay**			
Total hospital stay (days)	6.1 ± 5.7	7.8 ± 6.1	0.001 **
Postoperative hospital stay (days)	4.0 ± 1.4	4.4 ± 1.8	0.002 **
Re-admission	4 (0.5)	3 (1.8)	0.108
ICU admission	3 (0.4)	0 (0.0)	1.000
**Total**	42 (5.3)	20 (11.8)	0.003 **
Respiratory complications	9 (1.1)	6 (3.6)	0.033 *
Ileus	6 (0.8)	3 (1.8)	0.199
Bleeding	10 (1.3)	2 (1.2)	1.000
Fever	10 (1.3)	2 (1.2)	1.000
Need for antihypertensive	3 (0.4)	5 (2.9)	0.006 **
Others	9 (1.1)	8 (4.7)	0.004 **

Values are presented as number (percentage) or mean ± standard deviation. * *p* < 0.05, ** *p* < 0.01.

**Table 4:** Comparison of perioperative characteristics between patients with and without postoperative respiratory complications.

	No Respiratory Complications (n = 944)	Respiratory Complications (n = 15)	*p*-Value
**Preoperative characteristics**			
Pre-eclampsia	163 (17.3)	6 (40.0)	0.034 *
Weight (kg)	75.1 ± 32.1	74.9 ± 18.3	0.983
DM	137 (14.5)	3 (20.0)	0.470
Respiratory disease	22 (2.3)	1 (6.7)	0.307
Hemoglobin (g/dL)	11.6 ± 1.6	11.7 ± 1.4	0.928
PT (INR)	0.9 ± 0.1	0.9 ± 0.1	0.084
Albumin	3.6 ± 1.4	3.2 ± 0.7	0.310
AST	19.2 ± 27.7	17.0 ± 10.0	0.760
BUN	9.4 ± 6.6	20.1 ± 27.1	0.148
Creatinine	0.6 ± 0.6	0.7 ± 0.3	0.446
**Intraoperative factors**			
Emergency operation	582	11	0.431
Estimated blood loss (mL)	448.3 ± 268.8	376.9 ± 101.3	0.339
Infused Crystalloid (mL)	754.3 ± 482.8	650.0 ± 315.6	0.438
**Postoperative outcomes**			
ICU	1 (0.1)	2 (13.3)	0.001 **
fever	11 (1.2)	1 (6.7)	0.173
Time to discharge (days)	4.0 ± 1.4	5.7 ± 3.6	0.086

Values are presented as mean ± standard deviation (SD) or number (percentage). * *p* < 0.05, ** *p* < 0.01.

## Data Availability

The data presented in this study are available upon reasonable request from the corresponding author. The data are not publicly available because of institutional privacy regulations.

## References

[1] American College of Obstetricians and Gynecologists (2020). Gestational Hypertension and Preeclampsia: ACOG Practice Bulletin, Number 222. Obstet. Gynecol..

[2] Jeyabalan A. (2007). Preeclampsia: A perturbation of the maternal-fetal balance?. AMA J. Ethics.

[3] Rana S., Lemoine E., Granger J.P., Karumanchi S.A. (2019). Preeclampsia: Pathophysiology, Challenges, and Perspectives. Circ. Res..

[4] Phipps E.A., Thadhani R., Benzing T., Karumanchi S.A. (2019). Pre-eclampsia: Pathogenesis, novel diagnostics and therapies. Nat. Rev. Nephrol..

[5] Ives C.W., Sinkey R., Rajapreyar I., Tita A.T.N., Oparil S. (2020). Preeclampsia-Pathophysiology and Clinical Presentations: JACC State-of-the-Art Review. J. Am. Coll. Cardiol..

[6] Szpera-Gozdziewicz A., Breborowicz G.H. (2014). Endothelial dysfunction in the pathogenesis of pre-eclampsia. Front. Biosci. (Landmark Ed.).

[7] Cheng C., Liao A.H., Chen C.Y., Lin Y.C., Kang Y.N. (2021). A systematic review with network meta-analysis on mono strategy of anaesthesia for preeclampsia in caesarean section. Sci. Rep..

[8] Dennis A.T., Castro J., Carr C., Simmons S., Permezel M., Royse C. (2012). Haemodynamics in women with untreated pre-eclampsia. Anaesthesia.

[9] Pasokpuckdee K., Boriboonhirunsarn D. (2023). Incidence of Preeclampsia and Cesarean Section Rate According to the Robson Classification. Cureus.

[10] Neme D., Aweke Z., Jemal B., Mulgeta H., Regasa T., Garolla G., Zemedkun A., Sintayhu A. (2022). Effect of anesthesia choice on hemodynamic stability and fetomaternal outcome of the preeclamptic patient undergoing cesarean section. Ann. Med. Surg..

[11] Ankichetty S.P., Chin K.J., Chan V.W., Sahajanandan R., Tan H., Grewal A., Perlas A. (2013). Regional anesthesia in patients with pregnancy induced hypertension. J. Anaesthesiol. Clin. Pharmacol..

[12] Ring L., Landau R., Delgado C. (2021). The Current Role of General Anesthesia for Cesarean Delivery. Curr. Anesthesiol. Rep..

[13] Kaur H., Kolli M. (2021). Acute Pulmonary Edema in Pregnancy—Fluid Overload or Atypical Pre-eclampsia. Cureus.

[14] Heitkamp A., Sandberg E., Moodley A., Burke J., van Roosmalen J., Gebhardt S., Vollmer L., de Vries J.I., van den Akker T., Theron G. (2023). Pulmonary oedema in the course of severe maternal outcome in South Africa: A cohort study combined with clinical audit. Trop. Med. Int. Health.

[15] Dennis A.T. (2012). Management of pre-eclampsia: Issues for anaesthetists. Anaesthesia.

[16] Aregawi A., Terefe T., Admasu W., Akalu L. (2018). Comparing the Effect of Spinal and General Anaesthesia for Pre-Eclamptic Mothers Who Underwent Caesarean Delivery in A Tertiary, Addis Ababa, Ethiopia. Ethiop. J. Health Sci..

[17] Chattopadhyay S., Das A., Pahari S. (2014). Fetomaternal outcome in severe preeclamptic women undergoing emergency cesarean section under either general or spinal anesthesia. J. Pregnancy.

[18] Ozden M.G.N., Koruk S., Collak Z., Panik N. (2023). Comparison of the effects of general and spinal anesthesia for cesarean delivery on maternal and fetal outcomes: A retrospective analysis of data. North. Clin. Istanb..

[19] Unal B.S., Dennis A.T. (2023). Perioperative Complications in Patients with Preeclampsia Undergoing Caesarean Section Surgery. J. Clin. Med..

[20] Okafor U.V., Okezie O. (2005). Maternal and fetal outcome of anaesthesia for caesarean delivery in preeclampsia/eclampsia in Enugu, Nigeria: A retrospective observational study. Int. J. Obstet. Anesth..

[21] Canet J., Gallart L., Gomar C., Paluzie G., Vallès J., Castillo J., Sabaté S., Mazo V., Briones Z., Sanchis J. (2010). Prediction of postoperative pulmonary complications in a population-based surgical cohort. Anesthesiology.

[22] Hedenstierna G., Edmark L. (2005). The effects of anesthesia and muscle paralysis on the respiratory system. Intensive Care Med..

[23] Phipps E., Prasanna D., Brima W., Jim B. (2016). Preeclampsia: Updates in Pathogenesis, Definitions, and Guidelines. Clin. J. Am. Soc. Nephrol..

[24] Vandenbroucke J.P., von Elm E., Altman D.G., Gøtzsche P.C., Mulrow C.D., Pocock S.J., Poole C., Schlesselman J.J., Egger M. (2007). Strengthening the Reporting of Observational Studies in Epidemiology (STROBE): Explanation and elaboration. PLoS Med..

[25] Apfelbaum J.L., Hawkins J.L., Agarkar M., Bucklin B.A., Connis R.T., Gambling D.R., Mhyre J., Nickinovich D.G., Sherman H., Tsen L.C. (2016). Practice Guidelines for Obstetric Anesthesia: An Updated Report by the American Society of Anesthesiologists Task Force on Obstetric Anesthesia and the Society for Obstetric Anesthesia and Perinatology. Anesthesiology.

[26] Bauer M.E., Arendt K., Beilin Y., Gernsheimer T., Perez Botero J., James A.H., Yaghmour E., Toledano R.D., Turrentine M., Houle T. (2021). The Society for Obstetric Anesthesia and Perinatology Interdisciplinary Consensus Statement on Neuraxial Procedures in Obstetric Patients With Thrombocytopenia. Anesth. Analg..

[27] Girard T., Savoldelli G.L. (2024). Failed spinal anesthesia for cesarean delivery: Prevention, identification and management. Curr. Opin. Anaesthesiol..

[28] Smetana G.W., Lawrence V.A., Cornell J.E. (2006). Preoperative pulmonary risk stratification for noncardiothoracic surgery: Systematic review for the American College of Physicians. Ann. Intern. Med..

[29] Qaseem T. (2006). Risk assessment for and strategies to reduce perioperative pulmonary complications. Ann. Intern. Med..

[30] Suhas S., Manjunatha N., Kumar C.N., Benegal V., Rao G.N., Varghese M., Gururaj G. (2023). Firth’s penalized logistic regression: A superior approach for analysis of data from India’s National Mental Health Survey, 2016. Indian J. Psychiatry.

[31] Firth D. (1993). Bias reduction of maximum likelihood estimates. Biometrika.

[32] Heinze G., Schemper M. (2002). A solution to the problem of separation in logistic regression. Stat. Med..

[33] Redman C.W., Sargent I.L. (2005). Latest advances in understanding preeclampsia. Science.

[34] Laresgoiti-Servitje E. (2013). A leading role for the immune system in the pathophysiology of preeclampsia. J. Leukoc. Biol..

[35] Goulopoulou S., Davidge S.T. (2015). Molecular mechanisms of maternal vascular dysfunction in preeclampsia. Trends Mol. Med..

[36] Roberts J.M., Hubel C.A. (2009). The two stage model of preeclampsia: Variations on the theme. Placenta.

[37] Schobel H.P., Fischer T., Heuszer K., Geiger H., Schmieder R.E. (1996). Preeclampsia—A state of sympathetic overactivity. N. Engl. J. Med..

[38] Allen R.W., James M.F., Uys P.C. (1991). Attenuation of the pressor response to tracheal intubation in hypertensive proteinuric pregnant patients by lignocaine, alfentanil and magnesium sulphate. Br. J. Anaesth..

[39] Bansal S., Pawar M. (2002). Haemodynamic responses to laryngoscopy and intubation in patients with pregnancy-induced hypertension: Effect of intravenous esmolol with or without lidocaine. Int. J. Obstet. Anesth..

[40] Dyer R.A., Piercy J.L., Reed A.R., Lombard C.J., Schoeman L.K., James M.F. (2008). Hemodynamic changes associated with spinal anesthesia for cesarean delivery in severe preeclampsia. Anesthesiology.

[41] Goes A.S., Oliveira A.S., Silva R.O.S., da Fonseca F.L., Viana Oliveira C.R., de Lyra Junior D.P., de Oliveira-Filho A.D. (2025). Factors related to postpartum length of stay in women with pre-eclampsia: A systematic review. BMC Pregnancy Childbirth.

[42] Wen T., Yu V.X., Wright J.D., Goffman D., Attenello F., Mack W.J., D’Alton M., Friedman A.M. (2020). Postpartum length of stay and risk for readmission among women with preeclampsia. J. Matern. Fetal Neonatal Med..

[43] Ngene N.C., Moodley J. (2022). Fatal pulmonary oedema associated with severe pre-eclampsia: Challenges and lessons. Cardiovasc. J. Afr..

[44] Muthyala T., Mehrotra S., Sikka P., Suri V. (2016). Maternal Cardiac Diastolic Dysfunction by Doppler Echocardiography in Women with Preeclampsia. J. Clin. Diagn. Res..

[45] Melchiorre K., Sutherland G.R., Baltabaeva A., Liberati M., Thilaganathan B. (2011). Maternal cardiac dysfunction and remodeling in women with preeclampsia at term. Hypertension.

[46] Chen H., Tao F., Fang X., Wang X. (2016). Association of hypoproteinemia in preeclampsia with maternal and perinatal outcomes: A retrospective analysis of high-risk women. J. Res. Med. Sci..

[47] Bhatia R.K., Bottoms S.F., Saleh A.A., Norman G.S., Mammen E.F., Sokol R.J. (1987). Mechanisms for reduced colloid osmotic pressure in preeclampsia. Am. J. Obstet. Gynecol..

[48] Timokhina E., Kuzmina T., Strizhakov A., Pitskhelauri E., Ignatko I., Belousova V. (2019). Maternal Cardiac Function after Normal Delivery, Preeclampsia, and Eclampsia: A Prospective Study. J. Pregnancy.

[49] Wallace D.H., Leveno K.J., Cunningham F.G., Giesecke A.H., Shearer V.E., Sidawi J.E. (1995). Randomized comparison of general and regional anesthesia for cesarean delivery in pregnancies complicated by severe preeclampsia. Obstet. Gynecol..

[50] Sobhy S., Dharmarajah K., Arroyo-Manzano D., Navanatnarajah R., Noblet J., Zamora J., Thangaratinam S. (2017). Type of obstetric anesthesia administered and complications in women with preeclampsia in low- and middle-income countries: A systematic review. Hypertens. Pregnancy.

[51] Sibai B.M. (2003). Diagnosis and management of gestational hypertension and preeclampsia. Obstet. Gynecol..

[52] Zhang J., Meikle S., Trumble A. (2003). Severe maternal morbidity associated with hypertensive disorders in pregnancy in the United States. Hypertens. Pregnancy.

